# Strategies for power calculations in predictive biomarker studies in survival data

**DOI:** 10.18632/oncotarget.12124

**Published:** 2016-09-19

**Authors:** Dung-Tsa Chen, Po-Yu Huang, Hui-Yi Lin, Eric B. Haura, Scott J. Antonia, W. Douglas Cress, Jhanelle E. Gray

**Affiliations:** ^1^ Department of Biostatistics and Bioinformatics, Moffitt Cancer Center & Research Institute, Tampa, FL, USA; ^2^ Computational Intelligence Technology Center, Industrial Technology Research Institute, Taichung City, Taiwan; ^3^ Biostatistics program, School of Public Health, Louisiana State University Health Sciences Center , New Orleans, LA, USA; ^4^ Department of Thoracic Oncology, H. Lee Moffitt Cancer Center & Research Institute, Tampa, FL, USA; ^5^ Department of Molecular Oncology, H. Lee Moffitt Cancer Center & Research Institute, Tampa, FL, USA

**Keywords:** predictive biomarker, precision medicine, prospective study, retrospective study, survival data

## Abstract

**Purpose:**

Biomarkers and genomic signatures represent potentially predictive tools for precision medicine. Validation of predictive biomarkers in prospective or retrospective studies requires statistical justification of power and sample size. However, the design of these studies is complex and the statistical methods and associated software are limited, especially in survival data. Herein, we address common statistical design issues relevant to these two types of studies and provide guidance and a general template for analysis.

**Methods:**

A statistical interaction effect in the Cox proportional hazards model is used to describe predictive biomarkers. The analytic form by Peterson et al. and Lachin is utilized to calculate the statistical power for both prospective and retrospective studies.

**Results:**

We demonstrate that the common mistake of using only Hazard Ratio's Ratio (HRR) or two hazard ratios (HRs) can mislead power calculations. We establish that the appropriate parameter settings for prospective studies require median survival time (MST) in 4 subgroups (treatment and control in positive biomarker, treatment and control in negative biomarker). For the retrospective study which has fixed survival time and censored status, we develop a strategy to harmonize the hypothesized parameters and the study cohort. Moreover, we provide an easily-adapted R software application to generate a template of statistical plan for predictive biomarker validation so investigators can easily incorporate into their study proposals.

**Conclusion:**

Our study provides guidance and software to help biostatisticians and clinicians design sound clinical studies for testing predictive biomarkers.

## INTRODUCTION

Precision medicine, or personalized medicine, aims to effectively treat patients and prevent disease and is rapidly evolving, as evidenced by recent precision medicine initiatives and the US “Moonshot” (www.nih.gov/precisionmedicine and http://www.cancer.gov/research/key-initiatives/moonshot-cancer-initiative). In cancer research, various studies have identified potential predictive biomarkers to help physicians determine which treatment will result in the best outcome for a given patient [1-9]. For example, somatic mutations in the epidermal growth factor receptor (*EGFR*) gene predict which patients with non-small-cell lung carcinoma (NSCLC) will likely respond to gefitinib or erlotinib treatment [1, 3, 9]. For breast cancer, Oncotype DX, a gene signature, provides risk assignments in patients whose disease risk is undetermined by routine clinical variables [8]. In order to claim a predictive biomarker, validation using independent retrospective cohorts and/or prospective clinical trials is necessary [10]. However, such studies require rigorous sample size justification to indicate whether the proposed cohorts have sufficient power. Unfortunately, there is a paucity of literature on this topic due to the complexity of predictive models, especially in survival data [11-19]. From the statistical point of view, predictive models can be translated into the statistical interaction effect model by examining the interaction term to assess the differential treatment effect when the biomarker status changes. For survival data, a few methods based on Cox proportional hazards (PH) model have been used to calculate power and sample size for the interaction effect [11-13, 20]. However, utilization of these methods is not always straightforward due to the complexity of parameter settings.

In this study, we evaluate a series of key issues often ignored in power calculations for the interaction effect in survival data, such as hazard ratio (HR), hazard ratio's ratio (HRR), median survival time (MST), and censoring rate. Since prospective and retrospective studies have different design parameters, these issues are discussed individually.

## RESULTS

Power calculation for validation of predictive biomarker is illustrated in prospective and retrospective studies separately due to different design nature. MST is used for demonstration because of its common use in survival analysis.

### Prospective study

In prospective predictive biomarker validation studies, investigators often consider HRR (often derived from preliminary data) to be a sufficient parameter to calculate power or sample size. Here we highlight the sole use of HRR to calculate statistical power could be misleading. Similarly, the HR of treatment versus control in positive biomarker and the HR in the negative biomarker are insufficient to calculate power. In fact, HRR requires MST in the 4 subgroups (Table 1) in order to appropriately calculate the statistical power. Below are two examples to point out inappropriate calculation if HRR or two HRs are used. We also explain the causes and provide appropriate guidance for the prospective study.

**Table 1:** Median survival time in each subgroup

		Median survival time (MST)		Survival probability (S(t))	
		TREATMENT		TREATMENT	
		no	yes	no	yes
BIOMARKER	low (negative)	MST_(negative, control)_	MST_(negative, treatment)_	*S(t)*_(negative, control)_	*S(t)*_(negative, treatment)_
	high (positive)	MST_(positive, control)_	MST_(positive, treatment)_	*S(t)*_(positive, control)_	*S(t)*_(positive, treatment)_
		HRR=MST_(negative, treatment)_× MST_(positive, control)_)/ (MST_(negative, control)_× MST_(positive, treatment)_)		HRR= (*S(t)*_(negative, control)_× *S(t)*_(positive, treatment)_) /(*S(t)*_(negative, treatment)_× *S(t)*_(positive, control)_)	

Prior to power calculation, we specify additional parameters a-b to determine subgroup proportion in Table 2 and c-d to calculate subgroup censoring rate): (a) Ratio of treatment and control = (1:1), (b) Prevalence of the positive biomarker in the control and treatment groups, respectively = (0.5, 0.5), (c) Survival time distribution: Exponential distribution, (d) Censoring time distribution: Uniform distribution with 1 year follow-up and a total study time of 5 years, (e) Total sample size (*n* = 300), and (f) A two-sided 5% type I error.

**Table 2:** Proportion in each subgroup

		TREATMENT	
	Proportion	no	yes
BIOMARKER	low (negative)	γ_1,1_=(1-p_pos|treatment_) ×(1-p_treatment_)	γ_1,2_=(1-p_pos|treatment_) ×p_treatment_
	high (positive)	γ_2,1_=p_pos|treatment_ ×(1-p_treatment_)	γ_2,2_=p_pos|treatment_ ×p_treatment_

• Issue of HRR: There are many ways leading to the same HRR. Here we use a HRR of 4/9 as an example with two cases represented by different MSTs in Table 3. In Case 1A, MST is 4 years with treatment and 3 years without treatment in the negative biomarker group. For the positive biomarker group, MST is 3 years and 1 year with and without treatment, respectively. Thus, HR of treatment (compared to control) is 1/3 and 3/4 in the positive and negative biomarker groups, respectively. The corresponding HRR for the interaction effect is (1/3)/(3/4) = 4/9. In Case 1B, the negative biomarker group has a MST of 2 years with treatment and 1 year without treatment, while the positive biomarker group has a MST of 9 years if treatment is given and 2 years if not. The corresponding HRR for the interaction effect remains 4/9.

With these settings, we are able to calculate power using Equation 1 (Methods Section). We first calculate each subgroup censoring rate by comparing each subgroup MST to the uniform censoring time distribution through Equation 2. Results in Table 3 show that the censoring rate in the 4 subgroups ranges 17%-61% in Case 1A and 17%-80% in Case 1B. By plugging the resulting censoring rates and prevalence of each subgroup in the subgroup factor, $\sum 1/\left(\left(1-c_{i,j}\right)\times \gamma_{i,j}\right)$ in Equation 1 (i.e., summation of 1/( (1-censoring rate) × proportion of each subgroup)), Case 1A has a smaller subgroup factor of 31.74 compared to 37.7 in Case 1B. As a result, power is higher in Case 1A (71%) than in Case 1B (63%).

Issue of two HRs: Case 1A and 1C have the same HRR of 4/9 and are used for demonstration (Table 3). Case 1C has the same MSTs as Case 1A in the negative biomarker group with and without treatment. But the MSTs are double for Case 1C in the positive biomarker group (2 and 6 years in control and treatment group, respectively). Both cases have the same HRs in negative and positive biomarker groups (3/4 and 1/3, respectively). The only difference is that MSTs in the positive biomarker group are larger in Case 1C than in Case 1A. This difference leads to a higher subgroup censoring rate in Case 1C (38%-71%) than in Case 1A (17%-61%) in Table 3. Therefore, the power becomes lower in Case 1C (61%) than in Case 1A (71%).

Both examples show an 8-10% power difference and highlight the importance of the two issues (sole use of HRR or two HRs) which could lead to dramatic change of statistical power. This question could be solved by specifying MST in the 4 subgroups (Table 1). The 4 specified subgroup MSTs enable to compete with the censoring time to generate the censoring rate for each subgroup, and therefore to calculate power.

Once the power is calculated, we face another challenge of how to describe the power calculation to justify the sample size. Simply reporting the power and listing the specified parameters, may not sufficiently communicate to readers how the power is computed. Below, we share an easily-adaptable template developed using R software (‘PowerPredictiveBiomarker’ R package in package installation of Supplementary materials) and we illustrate its functionality using a previously described gene expression signature.

**Table 3:** MST and censoring rate of Case 1A-1C

	Case 1A (HR=4/9) : 71% power				Case 1B (HR=4/9) : 63% power				Case 1C (HR=4/9): 61% power			
	MST		Censoring rate (Overall=45%)		MST		Censoring rate (Overall=43%)		MST		Censoring rate (Overall=55.5%)	
	TREATMENT		TREATMENT		TREATMENT		TREATMENT		TREATMENT		TREATMENT	
BIOMARKER	no	yes	no	yes	no	yes	no	yes	no	yes	no	yes
low (negative)	3 yrs	4 yrs	52%	61%	1 yr	2 yrs	17%	38%	3 yrs	4 yrs	52%	61%
high (positive)	1 yr	3 yrs	17%	52%	2 yrs	9 yrs	38%	80%	2 yr	6 yrs	38%	71%

### Data example

The malignancy-risk (MR) gene signature [21, 22] has been described as a predictive signature that can identify early-stage NSCLC patients most likely to benefit from adjuvant chemotherapy (ACT). For example, in the JBR10 trial [21, 23] (a randomized two-arm trial), the MR signature shows a significant interaction effect (*p* = 0.02) with ACT treatment. Specifically, patients identified as high-risk by the MR signature tend to survive longer if they received ACT (*p* = 0.03). For low-risk patients, untreated patients had a longer survival but the result is not statistically significantly (*p* = 0.24). The estimated MST is 3.1 and 11.01 years for high-risk patients without and with ACT, respectively. For low-risk patients, the predicted MST is 10.11 and 6.66 years for patients without or with ACT, respectively.

Suppose we plan to validate the MR signature in a prospective study with a total sample size of 200, a ratio of 1:1 of treatment and control, a 50% prevalence of high MR in treatment and control, a 5-year study with 2 years of follow-up, and a two-sided 5% type I error. The statistical plan below provides power for sample size justification (details in Supplementary Figure 1).

Since patients with high MR have MST of 11.01 and 3.1 years in the treatment and control groups, respectively, the corresponding HR is 0.28 (3.1/11.01). In contrast, the HR is 1.52 (10.11/6.66) for patients with the low MR with the MSTs of 6.66 and 10.11 years in the treatment and control groups, respectively. Therefore, HRR of high versus low MR is 0.19 (0.28/1.52). Table 4A summarizes the MST for each subgroup. In addition, the overall MST (combination of the 4 subgroups) is also obtained as 6.98 years accordingly. Similarly, MSTs for the treatment, control, high MR, and low MR groups are 8.56, 5.6, 5.84, 8.21 years, respectively.

We also assume that (1) the percentage of patients in the treatment group is 50%, and (2) the prevalence of high MR is 50% and 50% in the treatment and control groups, respectively. With both assumptions, the subgroup proportion ranges 25% to 25% in Table 4B. The sample size of subgroup is between 50 and 50 (Table 4C).

With a total study time of 5 years with 2 years of follow-up and assumption of uniform distribution for the censoring time, the censoring time follows a uniform distribution between 2 and 5 years. By comparing with MST in Table 4A (assuming exponential distribution for survival time), censoring rates for the 4 subgroups range from 0.47 to 0.8 through Equation 2 (Table 4D).

By taking all together for consideration with a two-sided 5% type I error, the sample size of 200 will have an 87% power to detect a HRR of 0.19.

**Table 4:** MST, proportion, sample size, and censoring rate for each subgroup in MR signature in a prospective study

Power=87%	A: MST (years)		B: Subgroup Proportion		C: Subgroup Sample Size		D: Subgroup Censoring Rate	
HRR=0.19	TREATMENT		TREATMENT		TREATMENT		TREATMENT	
MR	no	yes	no	yes	no	yes	no	yes
Low	10.11	6.66	0.25	0.25	50	50	0.79	0.7
High	3.10	11.01	0.25	0.25	50	50	0.47	0.8

### Retrospective study

Prospective study requires accrual time and follow-up time to construct a censoring time. Uniform distribution is a good choice in this case. With given MST in each subgroup to form HRR, overall and subgroup censoring rate could be computed by Equation 2. In contrast, retrospective study often has observed survival time and censored status collected. Thus overall MST and censoring rate are already determined. If survival time and censored status are also available to treatment and control groups, we will know the corresponding MSTs and censoring rates. So, accrual time and follow-up time become irrelevant for the retrospective study. Since the retrospective study has this unique property, we consider a strategy to have hypothesized parameters more realistically reflecting to the study cohort.

Our strategy: Suppose the biomarker of interest has demonstrated potential clinical relevance in preliminary data with estimated MSTs in the 4 subgroups (Table 1) and the corresponding HRR (effect size). We plan to validate the biomarker in a retrospective study. Two key issues need to be addressed: (a) how to specify MSTs in the 4 subgroups such that the overall MST is comparable to the study cohort (or comparable MSTs between the preliminary data and study cohort in the treatment and control groups if the MST is available for the treatment and control in the study cohort) and (b) how to build an appropriate censoring time distribution such that the overall censoring rate is comparable to the study cohort (or comparable censoring rate between the preliminary data and study cohort in the treatment and control groups if the censoring rate is available for the treatment and control in the study cohort). In short, the key to the two issues is how to make the hypothesized settings comparable to the study cohort.

To generate a comparable overall MST, we consider an approach that employs a common factor, *k*, to all 4 subgroup MSTs such that the overall MST is the same to the study cohort through Equation 4. A similar formula (Equation 5) for the factor, k_g_, (*g* = 1 and 2 for the control and treatment, respectively) could be applied to treatment and control groups if both MSTs are available in the study cohort. Thus, by providing overall MST of the study cohort (or MSTs for treatment and control), we are able to scale the 4 subgroup MSTs of the preliminary data to generate a matched overall MST (or matched MSTs for treatment and control).

To determine a matched overall censoring rate, we use exponential distribution for censoring time because the observed survival time in retrospective study is variable in contrast to prospective study which uses the uniform distribution for a fixed maximum of study time. With the property of exponential distribution in both survival and censoring time, we can identify appropriate overall censoring time distribution or censoring time distribution for treatment and control through Equation 6-7. Once the censoring time distribution is determined, we are able to calculate censoring rate for each subgroup. With the specified HRR and prevalence of each subgroup, the power can be easily calculated through Equation 1.

### Data example (MR continued; Supplementary Figure 2)

Suppose we also plan to validate the MR signature by utilizing archived tissue samples from a retrospective study, a phase III, randomized three-arm clinical trial, neo-adjuvant taxol/carboplatin hope (NATCH) [24]. For this study, we focus on two arms, control arm and ACT arm, with OS as the primary outcome. The control arm has 141 patients with a 55% censoring rate and a MST of 4.8 years while there are 129 patients in the ACT arm with a censoring rate of 64% and a MST of 7.8 years. We expect that 50% of the tissue samples (*n* = (141+129)/2 = 135) are available and would like to know if this sample size has sufficient power to test the effect size (HRR = 0.19) for the signature validation.

We first compare MST of the treatment (and control) between the preliminary data and the study cohort. Results show that for the treatment group, the preliminary data had a higher MST than the study cohort (8.56 versus 7.8 years; Table 5A for MST from preliminary data). For the control group, the preliminary data had a higher MST than the study cohort (5.6 versus 4.8 years; Table 5A). To make both data comparable, we use Equation 5 for the factor, *k*_g_, to rescale the preliminary data (Table 5B for scaled MST). Since the formula need the proportion of each subgroup, we assume a 50% prevalence of positive biomarker in both treatment and control groups. Combined with 48% patients in the treatment group, the subgroup proportion ranges 24% to 26% with the sample size between 32 and 35 (Table 5C-D for proportion and sample size). By plugging the subgroup proportions in the formula, the resulting scale factor becomes 0.91 and 0.86 for the treatment and control, respectively. The rescaled data show that MSTs in treatment and control match well to the ones in the study cohort while retaining the same HRR.

The next step is to identify the censoring time distribution for the treatment and control groups in order to match the censoring rate to the study cohort. With the fixed censoring rate in the study cohort (64% and 55% for the treatment and control, respectively), subgroup scaled MST, and subgroup proportion (Table 5B-C), Equation 7 yields exponential censoring time distribution with MST 13.98 and 5.99 years for the treatment and control groups, respectively. As a result, censoring rate of each subgroup ranges from 0.41 to 0.70 in Table 5E by Equation 3.

Therefore, by taking all together for consideration (censoring rate and proportion for each subgroup) with a two-sided 5% type I error, the sample size of 135 will have 85% power to detect a HRR of 0.19 by Equation 1.

**Table 5:** MST, proportion, sample size, and censoring rate for each subgroup in MR signature in a retrospective study

	A: MST (years; from preliminary data)		B: MST (years; after scaled)		C: Subgroup Proportion		D: Subgroup Sample Size		E: Subgroup Censoring Rate	
	TREATMENT		TREATMENT		TREATMENT		TREATMENT		TREATMENT	
MR	no	yes	no	yes	no	yes	no	yes	no	yes
Low	10.11	6.66	8.69	6.06	0.26	0.24	35	32	0.41	0.70
High	3.10	11.01	2.67	10.02	0.26	0.24	35	32	0.69	0.58
Overall	5.6	8.56	4.82	7.80						

## DISCUSSION

Predictive biomarkers are potentially powerful tools for precision medicine. However, development of predictive biomarker requires prospective or retrospective studies for validation and solid statistical plans to justify sample size. Unfortunately, these studies have complex designs and the statistical methods and associated software are limited. In this study, we clarify common misconceptions in sole use of HRR and HRs, explain key parameters, and provide an easily-adapted statistical plan template for study design and power calculations. We highlight important differences between retrospective and prospective studies and our strategy to mimic study cohorts for proper power calculations.

In particular, for prospective studies, we highlight the need for MST in the 4 subgroups of Table 1. The use of only HRR or two HRs could mislead the power. Proper power calculation also requires enrollment time and follow-up time to define the censoring time in order to determine the censoring rate. In addition, we have to provide the prevalence of positive biomarker for treatment and control groups along with the ratio of treatment and control to determine the subgroup proportion. Once these parameters are well defined, power can be calculated for a given sample size and type I error through Equation 1. More importantly, based on our collaborative research experiences, we provide a template of statistical plan to detail derivation of censoring rate, proportion, and sample size for each subgroup, and therefore power calculation. To make the template accessible, we have developed a R software application (‘PowerPredictiveBiomarker’ R package) for the biomedical community.

For the retrospective study, we point out its distinct design which requires special treatment to generate appropriate power. Specifically, the retrospective study has fixed survival time and censored status (i.e., survival data have been collected). This feature challenges the hypothesized parameters. For example, suppose the preliminary data for discovering the biomarker has an overall MST of 6 years, but the study cohort you plan to validate has an overall MST of 2 years. Then both studies have a different overall MST. As a result, direct plugin of the preliminary results into the study cohort becomes problematic. Our strategy provides an approach to harmonize both studies such that their MSTs and censoring rates are comparable. Like the prospective study, the ‘PowerPredictiveBiomarker’ R package generates a template of statistical plan so investigators could easily incorporate into their retrospective study proposal. Example data from the published MR signature gives a hand-on experience for end-user to prepare the statistical plan for predictive biomarker validation.

In summary, power calculation for predictive biomarker in survival data requires well thought out parameter settings. Our study provides guidance and software to help biostatisticians and clinicians conduct power calculation in order to design a scientifically solid validation cohort for testing predictive biomarker.

## MATERIALS AND METHODS

Interaction effect model for survival data: An interaction effect model is described below based on the Cox PH model. The model includes a biomarker variable (positive and negative) and a treatment variable (treatment and control), and the interaction term of the two variables as expressed in the Cox PH model:

$$h\left(t\right)=h_{0}\left(t\right)\times e^{\beta_{1}\times biomarker+\beta_{2}+treatment+\beta_{2}\times biomarker\times treatment}$$

where *h(t)* is a hazard of event at time *t* and *h*_0_(*t*) is the baseline hazard.

This model involves three parameters, β_1_, β_2_, and β_3_:

β_1_ represents the biomarker effect. A positive value of β_1_ indicates shorter survival for those with a positive biomarker than the ones with a negative biomarker.

β_2_ represents the treatment effect. A negative value of β_2_ indicates survival improved in treated patients compared to untreated patients.

β_3_ represents the interaction effect between biomarker and treatment, a differential treatment effect when the biomarker value changes.

The parameter of interest for a predictive biomarker is the interaction term, β_3_. β_3_ can be expressed as log(HRR) where HRR is the abbreviation of Hazard Ratio's Ratio (i.e., hazard ratio (HR) in positive biomarker/HR in negative biomarker). Power calculation for the interaction model includes two methods by Peterson et al. [11] and Lachin [20].

Formula of power calculaiton by Peterson et al. and Lachin [11, 20] methods: Both methods have a similar analytic form of power function to estimate the interaction effect, β_3_, with the formula below

(Eq.1) $${\text{Power}}_{\left(\text{peterson or Lachin}\right)}=\Phi \left(-{\text{Z}}_{1-\alpha /2}+\sqrt{\frac{N\times \log{\left(HRR\right)}^{2}}{\sum 1/\left(\left(1-c_{i,j}\right)\times \gamma_{i,j}\right)}}\right)$$

Where HRR could be expressed as exp(β_3_), Ф is a cumulative normal distribution, Z is the normal quantile at 1-α/2 level, N is the total sample size, *c_i,j_* is the censoring rate and *y_i,j_* is the proportion for subgroup i, j with i = 1 and 2 for negative and positive biomarker, respectively, and with j = 1 and 2 for control and treatment group, respectively.

Prospective and retrospective study for predictive biomarker validtaion: The prospective study we consider for the application is the biomarker stratified design which patients within each biomarker subgroup are randomly assigned to treatment or control groups [25-27]. One successful example is the ‘Marker Validation of Erlotinib in Lung Cancer’ (MARVEL) trial using EGFR gene to stratify patients and then randomly assign patients with erlotinib or pemetrexed (ClinicalTrials.gov ID: NCT00738881). The other potential design is a prospective observational trial which biomarker status is measured for each patient, but not used for treatment assignment, such as ‘A multi-center prospective observational biomarker study on egfrm+ non-small cell lung cancer patients with leptomeningeal metastasis’ (ClinicalTrials.gov ID: NCT02803619). In short, both designs allow us to validate if the biomarker is predictive to treatment in a prospective way. For the retrospective study, our focus is a study that could utilize achieved tissue samples from a randomized clinical trial (RCT). For example, JBR10, a complete RCT to evaluate chemotherapy, has been utilized to validating various gene signatures in NSCLC [21, 23, 28]. Another example is ‘Alliance for Clinical Trials in Oncology’ which allow biospecimen request from various complete RCTs.

Challenges: While the analytic form in Equation 1 is attractive, it has issues of how to get HRR for the predictive effect size, how to compute *c_i,j_* for the censoring rate, and how to calculate *γ_i,j_* for the subgroup proportion. For retrospective study, other unique issues are how to hypothesize MSTs in the 4 subgroups of Table 1 and how to identify appropriate censoring time distribution to better fit the study cohort. Below we provide guidance to address these problems.

HRR: By definition, HRR is a ratio of two hazard ratios between the positive and negative biomarker (negative biomarker as reference) where the hazard ratio is referred to the comparison of hazard rate (λ) between treatment and control (control as reference). Thus, HRR is composed of four subgroups (treatment and control in positive biomarker, treatment and control in negative biomarker; Table 1) and has the form, ( λ_(negative, treatment)_× λ _(positive, control)_)/ (λ _(negative, control)_× λ _(positive, treatment)_). While it is not straightforward to get the hazard rate, we find out two useful surrogates: median survival time (MST) and survival probability at a specific time point (*S*(*t*)). Both are summary statistics commonly used in survival analysis. With the assumption of exponential distribution for survival time, these summary statistics are exchangeable with the hazard rate, λ, under the following formula:

$$\lambda =\frac{\log\left(2\right)}{MST}=\frac{-\log\left(S\left(t\right)\right)}{t}$$

Therefore, HRR can be based upon MST or *S(t)* in the four subgroups of Table 1 (i.e., HRR = (MST_(negative, treatment)_× MST_(positive, control)_)/ (MST_(negative, control)_× MST(positive, treatment)) or = (*S(t)*_(negative, control)_× *S(t)*_(positive, treatment)_) /(*S(t)*_(negative, treatment)_× *S(t)*_(positive, control)_)).

Censoring rate: Since the analytic form (Equation 1) requires subgroup censoring rate (*c_i,j_*) and an overall censoring rate (c), the following equation' is used to calculate the censoring rate.

$$\begin{array}{l} c_{i,j}=\text{P}\left(C<T_{i,j}\right)=1-\text{P}\left(C>T_{i,j}\right)=1-\int_{0}^{\infty }\text{P}\left(T_{i,j}\leq t\right)f_{c}\left(\text{t}\right)\text{dt}\\ c=1-\sum \left[\gamma_{i,j}\times \int_{0}^{\infty }\text{P}\left(T_{i,j}\leq t\right)f_{c}\left(\text{t}\right)\text{dt}\right] \end{array}$$

where *T_i,j_* is the survival time of subgroup*_(i,j)_* (i = 1 and 2 for negative and positive biomarker, respectively, and with j = 1 and 2 for control and treatment group), and C is the censoring time with a density function, *f_c_(t)*.

When *T_i,j_* follows an exponential distribution with λ*_(i,j)_* or *MST_(i,,j)_*, the subgroup censoring rate (*c_i,j_*) could be simplified for a uniform censoring time or an exponential censoring time.

(Eq. 2) $$\begin{array}{l} c_{i,j}=\frac{1}{\left(b-a\right)\times \lambda_{i,j}}\left(e^{-a\lambda_{i,j}}-e^{-b\lambda_{i,j}}\right)\\ =\frac{1}{\left(b-a\right)\times \log\left(2\right)/MST_{i,j}}\left(e^{-a\times \log\left(2\right)/MST_{i,j}}-e^{-b\times \log\left(2\right)/MST_{i,j}}\right) \end{array}$$

for a uniform censoring time with two parameters: a and b as the corresponding lower and upper boundary.

(Eq. 3) $$c_{i,j}=\frac{\lambda_{i,j}}{\left(\lambda_{i,j}+\lambda_{c}\right)}=\frac{\frac{1}{MST_{i,j}}}{\left(\frac{1}{MST_{i,j}}+\frac{1}{MST_{c}}\right)}$$

for an exponential censoring time with λ*_c_*, or MST*_c_*.

Subgroup proportion: Calculation of *γ_i,j_* could be through the prevalence of positive biomarker in the treatment group (p_(pos|treatment}_) and in the control group ((p_(pos|control}_)) and the proportion of patients receiving treatment ((p_(treatment}_)) (Table 2). Specifically, the proportion of control group is γ_1,1_ = (1-p_pos|treatment_) ×(1-p_treatment_) for the negative biomarker and γ_2,1_ = p_pos|treatment_ ×(1-p_treatment_) for the positive biomarker. Similarly, the proportion of negative biomarker in the treatment group is γ_1,2_ = (1-p_pos|treatment_) ×p_treatment_. For the positive biomarker, the proportion is γ_2,2_ = p_pos|treatment_ ×p_treatment_.

Comparable overall MST: One key issue in retrospective study is how to hypothesize MSTs in the 4 subgroups of Table 1 such that the overall MST is comparable to the study cohort. Our approach is to find a common factor, *k*, such that the weighted summation (subgroup proportion as weight) equates to 0.5 (definition of MST) by the formula:

(Eq. 4) $$\begin{array}{c} \lambda_{0}\sum_{i=1}^{2}\sum_{j=1}^{2}\frac{\gamma_{i,j}}{\left(\lambda_{0}+\frac{\lambda_{i,j}}{k}\right)}=0.5\text{ or}\\ \frac{1}{MST_{0}}\sum_{i=1}^{2}\sum_{j=1}^{2}\frac{\gamma_{i,j}}{\left(\frac{1}{MST_{0}}+\frac{1}{k\times MST_{i,j}}\right)}=0.5 \end{array}$$

where λ_i,j_ = *log(2)*/*MST_(i,j)_* is the hazard rate and γ*_i,j_* is the prevalence for the subgroup (i,j), and λ_0_ and *MST*_0_ are the overall hazard rate and overall MST, respectively, for the study cohort. The approach ensures the rescaled 4 subgroup MSTs have the same overall MST to the study cohort. Derivation of the formula is based on Equation 3. With the assumption of exponential distribution for two survival time distributions, we are able to compare each subgroup distribution with the overall distribution of the study cohort. Then with the common factor, *k*, the weighted summation over the 4 subgroups yields Equation 4.

A similar formula for the factor, *k*_g_, (*g* = 1 and 2 for the control and treatment, respectively) could be applied to treatment and control groups if both MSTs are available in the study cohort:

(Eq. 5) $$\begin{array}{c} \lambda_{0,g}\sum_{i=1}^{2}\frac{\gamma_{i,j}/\left(\gamma_{1,g}+\gamma_{2,g}\right)}{\left(\lambda_{0,g}+\frac{\lambda_{i,g}}{k_{g}}\right)}=0.5\text{ or}\\ \frac{1}{MST_{0,g}}\sum_{i=1}^{2}\frac{\gamma_{i,g}/\left(\gamma_{1,g}+\gamma_{2,g}\right)}{\left(\frac{1}{MST_{0,g}}+\frac{1}{k_{g}\times MST_{i,g}}\right)}=0.5 \end{array}$$

where λ_i,g_ = *log(2)/MST_(i,g)_* is the hazard rate of the subgroup (i,g) (i = 1 and 2 for positive and negative biomarker, respectively) in the treatment or control groups, and y*_i,g_* is the corresponding prevalence. λ_0,*g*_ and *MST*_0,*g*_ are the hazard rate and MST, respectively, for the control (*g* = 1) and the treatment (*g* = 2) in the study cohort.

Censoring time distribution for the retrospective study: Another key issue in retrospective study is to select an appropriate censoring time distribution such that the overall censoring rate is comparable to the study cohort. With the property of exponential distribution in both survival and censoring time through Equation 3, distribution of the overall censoring time can be identified through the formula:

(Eq. 6) $$\begin{array}{c} \lambda_{0}\sum_{i=1}^{2}\sum_{j=1}^{2}\frac{\gamma_{i,j}}{\left(\lambda_{c}+\lambda_{i,j}\right)}=1-c\text{ or}\\ \frac{1}{MST_{c}}\sum_{i=1}^{2}\sum_{j=1}^{2}\frac{\gamma_{i,j}}{\left(\frac{1}{MST_{c}}+\frac{1}{MST_{i,j}}\right)}=1-c \end{array}$$

where λ_c_ and *MST*_c_ are the hazard rate and MST, respectively, for the overall censoring time and *c* is the overall censoring rate. Similarly, the formula of censoring time distribution for the treatment and control is

(Eq. 7) $$\begin{array}{c} \lambda_{c,g}\sum_{i=1}^{2}\frac{\gamma_{i,g}/\left(\gamma_{1,g}+\gamma_{2,g}\right)}{\left(\lambda_{c,g}+\lambda_{i,g}\right)}=1-c_{g}\text{ or}\\ \frac{1}{MST_{c,g}}\sum_{i=1}^{2}\frac{\gamma_{i,g}/\left(\gamma_{1,g}+\gamma_{2,g}\right)}{\left(\frac{1}{MST_{c,g}}+\frac{1}{MST_{i,g}}\right)}=1-c_{g} \end{array}$$

where λ_c,g_ and *MST_c,g_* are the hazard rate and MST, respectively, of the censoring time and *c*_g_ is the censoring rate for control (*g* = 1) and treatment (*g* = 2).

## SUPPLEMENTARY MATERIALS


