# Supplementary material for: Strategies for power calculations in predictive biomarker studies in survival data
Source: Oncotarget. 2016 Sep 19;7(49):80373–81. doi: 10.18632/oncotarget.12124 (PMC5348326; doi:10.18632/oncotarget.12124)
Supplement: Supplementary file 1 [file oncotarget-07-80373-s001.pdf]

Strategies for power calculations in predictive biomarker studies in survival data

Supplementary Materials

Instruction of ‘PowerPredictiveBiomarker’ R package.

Supplementary Figure 1 for prospective study: (A) parameter settings, (B) result output in MR signature, and (C) statistical plan.

Supplementary Figure 2 for retrospective study: (A) parameter settings, (B) result output in MR signature, and (C) statistical plan

Instruction of ‘PowerPredictiveBiomarker’ R package

1. Installation: In command window of R studio, perform these commands:
   ```
   if (!require("devtools"))
     install.packages("devtools")
   devtools::install_github("dungtsa/PowerPredictiveBiomarker",force = TRUE)
   ```

2. Get started (in command window of R studio):
   ```
   require("PowerPredictiveBiomarker")
   PowerPredictiveBiomarker.shiny()
   ```

Supplementary Figure 1A: Parameter settings for prospective study

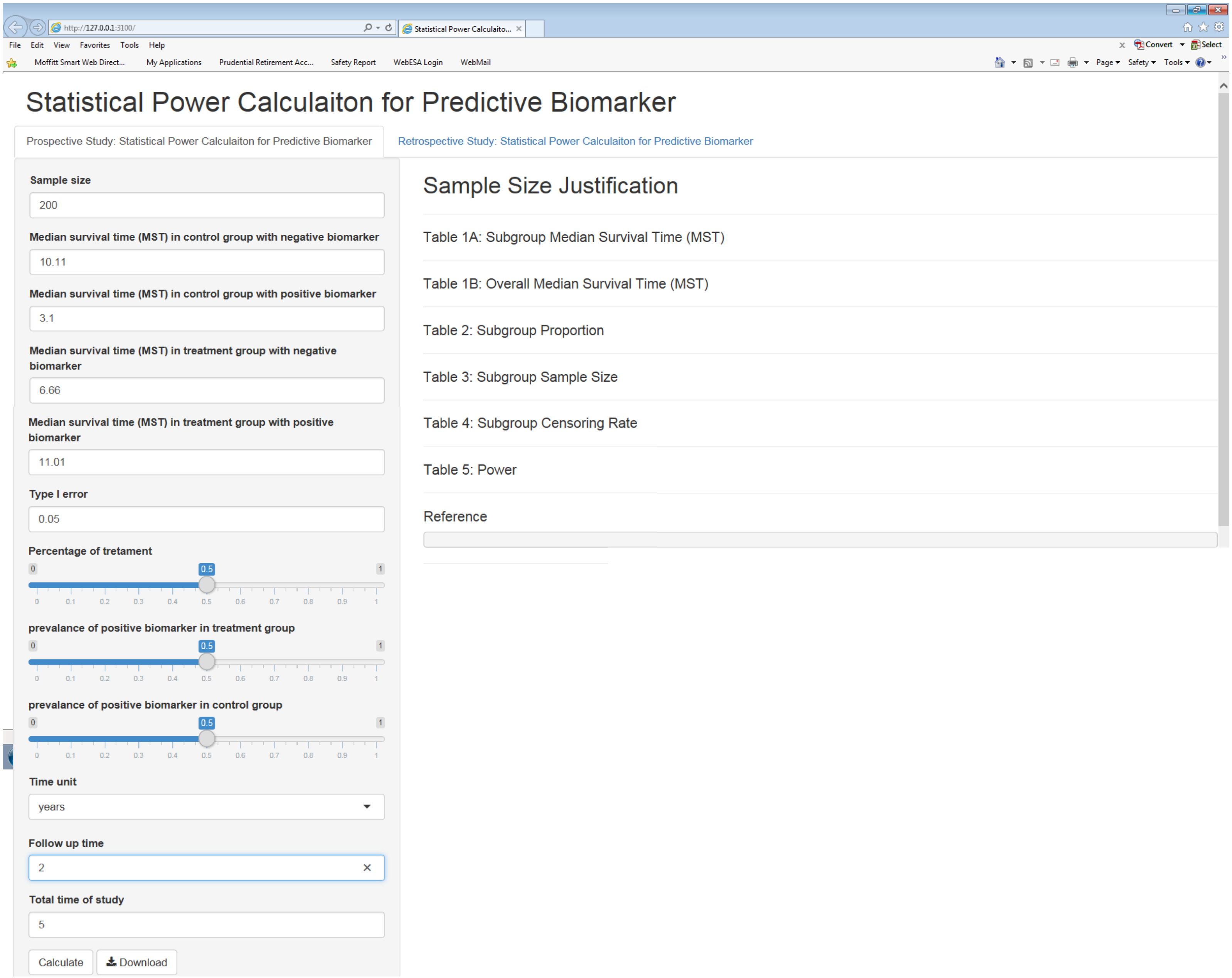

Supplementary Figure 1B: Result output for prospective study

Statistical Power Calculaiton for Predictive Biomarker

Prospective Study: Statistical Power Calculaiton for Predictive Biomarker | Retrospective Study: Statistical Power Calculaiton for Predictive Biomarker

Sample size

200

Median survival time (MST) in control group with negative biomarker

10.11

Median survival time (MST) in control group with positive biomarker

3.1

Median survival time (MST) in treatment group with negative biomarker

6.66

Median survival time (MST) in treatment group with positive biomarker

11.01

Type I error

0.05

Percentage of tretament

0 0.5 1

0 0.1 0.2 0.3 0.4 0.5 0.6 0.7 0.8 0.9 1

prevalance of positive biomarker in treatment group

0 0.5 1

0 0.1 0.2 0.3 0.4 0.5 0.6 0.7 0.8 0.9 1

prevalance of positive biomarker in control group

0 0.5 1

0 0.1 0.2 0.3 0.4 0.5 0.6 0.7 0.8 0.9 1

Time unit

years

Follow up time

2

Total time of study

5

Calculate | Download

Sample Size Justification

We plan to use a total sample size of 200 to validate the predictive effect of the biomarker. Justification of the sample size is based on the statistical interaction model with a biomarker variable (positive and negative), a treatment variable (treatment and control), and the interaction term of the two variables in the Cox proportional hazard model: $h(t)=h0(t) \times exp(\beta_1 \times biomarker+\beta_2 \times treatment+\beta_3 \times biomarker \times treatment)$ where $h(t)$ is a hazard at time t and $h0(t)$ is the baseline hazard. The goal is to test $H_0: \beta_3=0$ where β_3 can be expressed as the log scale of hazard ratio's ratio (HRR; described later). The methodology we used is from Peterson and Lachin methods (ref 1 and 2). Below is the justification of the sample size. We assume the median survival time (MST) is 11.01 and 3.1 years in the treatment and control groups, respectively, for patients with high (or positive) biomarker. The corresponding hazard ratio (HR) is 0.28. For patients with the low (or negative) biomarker, we assume their MSTs are 6.66 and 10.11 years in the treatment and control groups, respectively. The corresponding HR is 1.52. Therefore, the hazard ratio's ratio (HRR) of high versus low biomarker is 0.19. Table 1A summarizes the MST for each subgroup. In addition, the overall MST (combination of the 4 subgroups) is also obtained as 6.98 years accordingly (Table 1B). Similarly, MSTs for the treatment, control, positive, and negative groups are 8.56, 5.6, 5.84, 8.21 years, respectively. We also assume that (1) the percentage of patients in the treatment group is 50%, and (2) the prevalence of high biomarker is 50% and 50% in the treatment and control groups, respectively. With both assumptions, the subgroup proportion ranges 25% to 25% (Table 2). The sample size of subgroup is between 50 and 50 (Table 3). Total time of the study will be 5 years with 2 years of follow-up. Under the assumption of uniform distribution for the censoring time, the censoring time follows a uniform distribution between 2 and 5 years. By comparing with MST in Table 1A (assuming exponential distribution for survival time), we are able to calculate subgroup censoring rate. Table 4 lists censoring rate for each subgroup, ranging from 0.47 to 0.8. By taking all together for consideration with a two-sided 5% type I error, the sample size of 200 will have 87% power to detect a HRR of 0.19.

Table 1A: Subgroup Median Survival Time (MST)

	Control	Treatment	HR	HRR
Negative	10.11	6.66	1.52	0.19
Positive	3.10	11.01	0.28	

Table 1B: Overall Median Survival Time (MST)

	MST
overall	6.98
treatment	8.56
control	5.60
positive	5.84
negative	8.21

Table 2: Subgroup Proportion

	Control	Treatment
Negative	0.25	0.25
Positive	0.25	0.25

Table 3: Subgroup Sample Size

	Control	Treatment
Negative	50.00	50.00
Positive	50.00	50.00

Table 4: Subgroup Censoring Rate

	Control	Treatment
Negative	0.79	0.70
Positive	0.47	0.80

Table 5: Power

	Power
Peterson	0.87
Lachin	0.87

Supplementary Figure 1C: Statistical Plan

Statistical Power Calculation for Predictive Biomarker in Prospective Study

Chen et al.

==

Sample Size Justification

We plan to use a total sample size of 200 to validate the predictive effect of the biomarker. Justification of the sample size is based on the statistical interaction model with a biomarker variable (positive and negative), a treatment variable (treatment and control), and the interaction term of the two variables in the Cox proportional hazard model: $h(t) = h0(t) \times exp(\beta_1 \times biomarker + \beta_2 \times treatment + \beta_3 \times biomarker \times treatment)$ where $h(t)$ is a hazard at time t and $h0(t)$ is the baseline hazard. The goal is to test $H_0: \beta_3 = 0$ where β_3 can be expressed as the log scale of hazard ratio's ratio (HRR; described later). The methodology we used is from Peterson and Lachin methods (ref 1 and 2). Below is the justification of the sample size.

We assume the median survival time (MST) is 11.01 and 3.1 years in the treatment and control groups, respectively, for patients with high (or positive) biomarker. The corresponding hazard ratio (HR) is 0.28. For patients with the low (or negative) biomarker, we assume their MSTs are 6.66 and 10.11 years in the treatment and control groups, respectively. The corresponding HR is 1.52. Therefore, the hazard ratio's ratio (HRR) of high versus low biomarker is 0.19. Table 1A summarizes the MST for each subgroup. In addition, the overall MST (combination of the 4 subgroups) is also obtained as 6.98 years accordingly (Table 1B). Similarly, MSTs for the treatment, control, positive, and negative groups are 8.56, 5.6, 5.84, 8.21 years, respectively.

We also assume that (1) the percentage of patients in the treatment group is 50%, and (2) the prevalence of high biomarker is 50% and 50% in the treatment and control groups, respectively. With both assumptions, the subgroup proportion ranges 25% to 25% (Table 2). The sample size of subgroup is between 50 and 50 (Table 3).

Total time of the study will be 5 years with 2 years of follow-up. Under the assumption of uniform distribution for the censoring time, the censoring time follows a uniform distribution between 2 and 5 years. By comparing with MST in Table 1A (assuming exponential distribution for survival time), we are able to calculate subgroup censoring rate. Table 4 lists censoring rate for each subgroup, ranging from 0.47 to 0.8.

By taking all together for consideration with a two-sided 5% type I error, the sample size of 200 will have 87% power to detect a HRR of 0.19.

Table 1A: Subgroup MSTs

	Control	Treatment	HR	HRR
Negative	10.11	6.66	1.52	0.19
Positive	3.10	11.01	0.28	NA

Table 1B: Subgroup MSTs

	MST
overall	6.98
treatment	8.56
control	5.60
positive	5.84
negative	8.21

Table 2: Subgroup Proportions

	Control	Treatment
Negative	0.25	0.25
Positive	0.25	0.25

Table 3: Subgroup Sample Size

	Control	Treatment
Negative	50	50
Positive	50	50

Table 4: Subgroup Censoring Rate

	Control	Treatment
Negative	0.79	0.7
Positive	0.47	0.8

Table 5: Statistical Power

	Power
Peterson	0.87
Lachin	0.87

References

1. Peterson, B. and S.L. George, Sample size requirements and length of study for testing interaction in a 2 x k factorial design when time-to-failure is the outcome [corrected]. Control Clin Trials, 1993. 14(6): p. 511-22.

2. Lachin, J.M., Sample size and power for a logrank test and Cox proportional hazards model with multiple groups and strata, or a quantitative covariate with multiple strata. Stat Med, 2013. 32(25): p. 4413-25.

Supplementary Figure 2A: Parameter settings for retrospective study

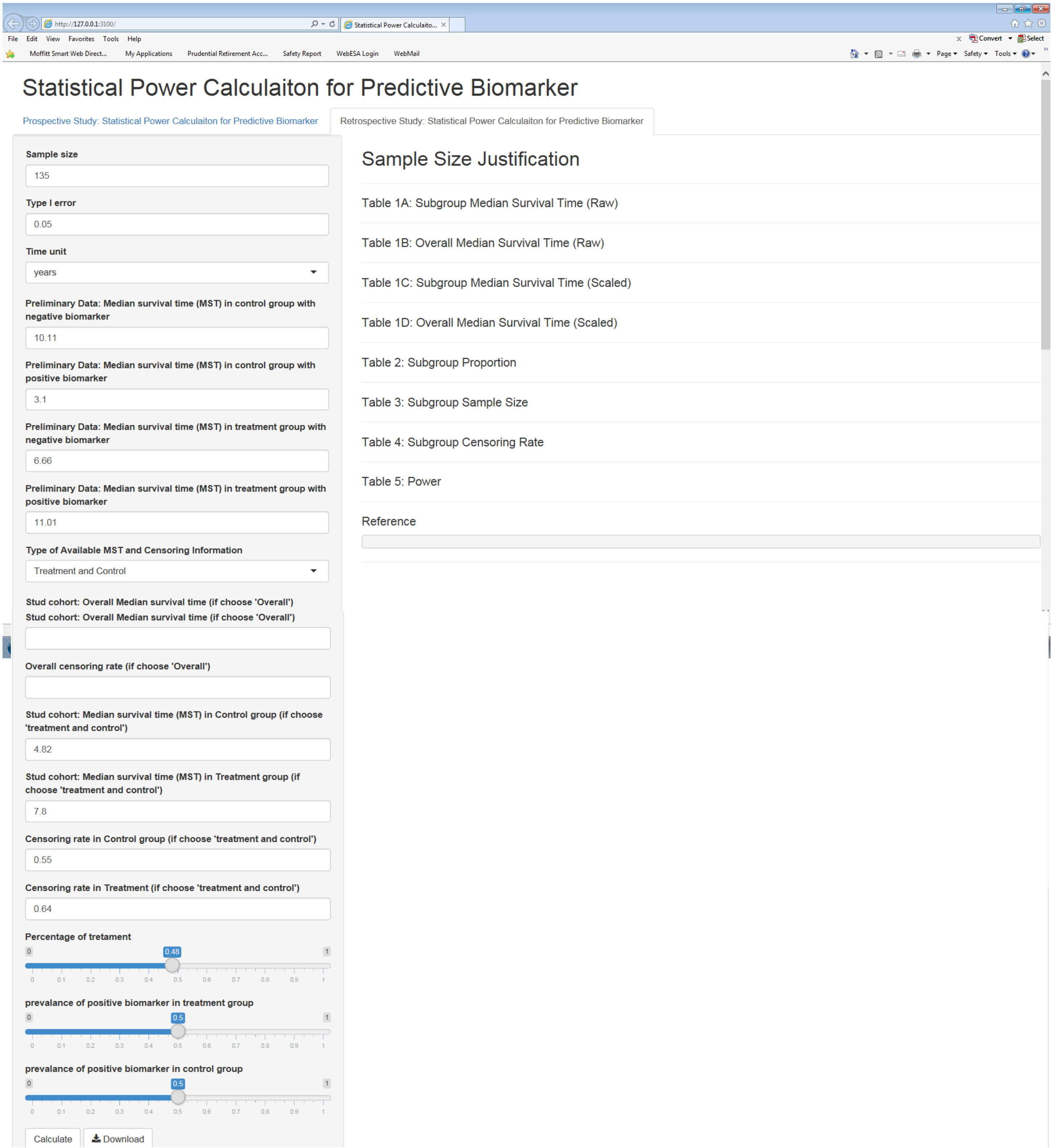

Supplementary Figure 2B: Result output for retrospective study

Statistical Power Calculaiton for Predictive Biomarker

Prospective Study: Statistical Power Calculaiton for Predictive Biomarker | Retrospective Study: Statistical Power Calculaiton for Predictive Biomarker

Sample size

135

Type I error

0.05

Time unit

years

Preliminary Data: Median survival time (MST) in control group with negative biomarker

10.11

Preliminary Data: Median survival time (MST) in control group with positive biomarker

3.1

Preliminary Data: Median survival time (MST) in treatment group with negative biomarker

6.66

Preliminary Data: Median survival time (MST) in treatment group with positive biomarker

11.01

Type of Available MST and Censoring Information

Treatment and Control

Stud cohort: Overall Median survival time (if choose 'Overall')

Overall censoring rate (if choose 'Overall')

Stud cohort: Median survival time (MST) in Control group (if choose 'treatment and control')

0 0.5 1

0 0.1 0.2 0.3 0.4 0.5 0.6 0.7 0.8 0.9 1

prevalance of positive biomarker in control group

0 0.5 1

0 0.1 0.2 0.3 0.4 0.5 0.6 0.7 0.8 0.9 1

Calculate | Download

Sample Size Justification

We plan to use a total sample size of 135 to validate the predictive effect of the biomarker. Justification of the sample size is based on the statistical interaction model with a biomarker variable (positive and negative), a treatment variable (treatment and control), and the interaction term of the two variables in the Cox proportional hazard model: $h(t)=h0(t) \times exp(\beta_1 \times biomarker+\beta_2 \times treatment+\beta_3 \times biomarker \times treatment)$ where $h(t)$ is a hazard at time t and $h0(t)$ is the baseline hazard. The goal is to test $H_0: \beta_3=0$ where β_3 can be expressed as the log scale of hazard ratio's ratio (HRR; described below). The methodology we used is from Peterson and Lachin methods (ref 1 and 2). Below is the justification of the sample size. Our preliminary data indicate the biomarker is able to detect an HRR of 0.19 (effect size) with a hazard ratio (HR) of 0.28 in the positive biomarker (11.01 and 3.1 years in the treatment and control groups, respectively) and a HR of 1.52 in the negative biomarker (6.66 and 10.11 years in the treatment and control groups, respectively) in Table 1A. The overall MST (combination of the 4 subgroups) is 6.92 years accordingly (Table 1B). Similarly, MSTs for the treatment and control groups are 8.56 and 5.6 years respectively. To apply the preliminary data to the study cohort to testing the effect size (HRR= 0.19) for the predictive biomarker, we compare MST of the treatment (and control) between the preliminary data and the study cohort. Results show that for the treatment group, the preliminary data had a higher MST than the study cohort (8.56 vs 7.8 years). For the control group, the preliminary data had a higher MST than the study cohort (5.6 vs 4.82 years). To make both data comparable, we rescale the preliminary data with a scale factor of 0.91 and 0.86 for the treatment and control, respectively. As a result, MSTs in treatment and control from Table 1 (after rescale; Table 1C) match well to the ones in the study cohort while retaining the same HRR. We also assume that (1) the percentage of patients in the treatment group is 48%, and (2) the prevalence of positive biomarker is 50% and 50% in the treatment and control groups, respectively. With both assumptions, the subgroup proportion ranges 24% to 26% (Table 2). The sample size of subgroup is between 32 and 35 (Table 3). Since the study cohort has a censoring rate of 0.64 and 0.55 for the treatment and control, respectively, in order to match the same censoring rates for Table 1C, the exponential distribution with MST of 13.98 and 5.99 was used for the censoring time in the treatment and control groups, respectively. Table 4 lists censoring rate for each subgroup, ranging from 0.41 to 0.7 By taking all together for consideration with a two-sided 5% type I error, the sample size of 135 will have 85% power to detect a HRR of 0.19.

Table 1A: Subgroup Median Survival Time (Raw)

	Control	Treatment	HR	HRR
C_low	10.11	6.66	1.52	0.19
C_high	3.10	11.01	0.28	

Table 1B: Overall Median Survival Time (Raw)

	MST
overall	6.92
treatment	8.56
control	5.60

Table 1C: Subgroup Median Survival Time (Scaled)

	Control	Treatment	HR	HRR
C low	8.69	6.06	1.43	0.19

Table 3: Subgroup Sample Size

	Control	Treatment
Negative	35.00	32.00
Positive	35.00	32.00

)

Table 4: Subgroup Censoring Rate

	Control	Treatment
Negative	0.41	0.70
Positive	0.69	0.58

Table 5: Power

	Power
Peterson	0.85
Lachin	0.85

Supplementary Figure 2C: Statistical Plan

Statistical Power Calculation for Predictive Biomarker in Retrospective Study

Chen et al.

==

Sample Size Justification

We plan to use a total sample size of 135 to validate the predictive effect of the biomarker. Justification of the sample size is based on the statistical interaction model with a biomarker variable (positive and negative), a treatment variable (treatment and control), and the interaction term of the two variables in the Cox proportional hazard model: $h(t) = h0(t) \times exp(\beta_1 \times biomarker + \beta_2 \times treatment + \beta_3 \times biomarker \times treatment)$ where $h(t)$ is a hazard at time t and $h0(t)$ is the baseline hazard. The goal is to test $H_0: \beta_3 = 0$ where β_3 can be expressed as the log scale of hazard ratio's ratio (HRR; described below). The methodology we used is from Peterson and Lachin methods (ref 1 and 2). Below is the justification of the sample size.

Our preliminary data indicate the biomarker is able to detect an HRR of 0.19 (effect size) with a hazard ratio (HR) of 0.28 in the positive biomarker (11.01 and 3.1 years in the treatment and control groups, respectively) and a HR of 1.52 in the negative biomarker (6.66 and 10.11 years in the treatment and control groups, respectively) in Table 1A. The overall MST (combination of the 4 subgroups) is 6.92 years accordingly (Table 1B). Similarly, MSTs for the treatment and control groups are 8.56 and 5.6 years respectively.

To apply the preliminary data to the study cohort to testing the effect size (HRR= 0.19) for the predictive biomarker, we compare MST of the treatment (and control) between the preliminary data and the study cohort. Results show that for the treatment group, the preliminary data had a higher MST than the study cohort (8.56 vs 7.8 years). For the control group, the preliminary data had a higher MST than the study cohort (5.6 vs 4.82 years). To make both data comparable, we rescale the preliminary data with a scale factor of 0.91 and 0.86 for the treatment and control, respectively. As a result, MSTs in treatment and control from Table 1 (after rescale; Table 1C) match well to the ones in the study cohort while retaining the same HRR.

We also assume that (1) the percentage of patients in the treatment group is 48%, and (2) the prevalence of positive biomarker is 50% and 50% in the treatment and control groups, respectively. With both assumptions, the subgroup proportion ranges 24% to 26% (Table 2). The sample size of subgroup is between 32 and 35 (Table 3).

Since the study cohort has a censoring rate of 0.64 and 0.55 for the treatment and control, respectively, in order to match the same censoring rates for Table 1C, the exponential distribution with MST of 13.98 and 5.99 was used for the censoring time in the treatment and control groups, respectively. Table 4 lists censoring rate for each subgroup, ranging from 0.41 to 0.7

By taking all together for consideration with a two-sided 5% type I error, the sample size of 135 will have 85% power to detect a HRR of 0.19.

Table 1A: Subgroup Median Survival Time (Raw)

	Control	Treatment	HR	HRR
C_low	10.11	6.66	1.52	0.19
C_high	3.10	11.01	0.28	NA

Table 1B: Overall Median Survival Time (Raw)

	MST
overall	6.92
treatment	8.56
control	5.60

Table 1C: Subgroup Median Survival Time (Scaled)

	Control	Treatment	HR	HRR
C_low	8.6946	6.0606	1.43	0.19
C_high	2.6660	10.0191	0.27	NA

Table 1D: Overall Median Survival Time (Scaled)

	MST
overall	6.116
treatment	7.796
control	4.816

Table 2: Subgroup Proportions

	Control	Treatment
Negative	0.26	0.24
Positive	0.26	0.24

Table 3: Subgroup Sample Size

	Control	Treatment
Negative	35	32
Positive	35	32

Table 4: Subgroup Censoring Rate

	Control	Treatment

Negative	0.41	0.70
Positive	0.69	0.58

Table 5: Statistical Power

	Power
Peterson	0.85
Lachin	0.85

References

1. Peterson, B. and S.L. George, Sample size requirements and length of study for testing interaction in a 2 x k factorial design when time-to-failure is the outcome [corrected]. Control Clin Trials, 1993. 14(6): p. 511-22.

2. Lachin, J.M., Sample size and power for a logrank test and Cox proportional hazards model with multiple groups and strata, or a quantitative covariate with multiple strata. Stat Med, 2013. 32(25): p. 4413-25.
